# Call for a Mass Convention of the Dentists

**Published:** 1880-07

**Authors:** J. Taft, R. Finley Hunt, R. B. Winder

**Affiliations:** General Chairman; Ch’n Com. So. Dental Ass’n.; Ch’n Com. of A. D. C.


Call for a Mass Convention of the Dentists of the United States to Organize a National Dental Association.—At their annual meetings in 1879, the Southern Dental Association and the American Dental Convention each appointed a committee invested with full power to adopt measures for the formation of a National Dental Association. The American Dental Association at its meeting in the same year appointed a committee to confer with these two committees on this movement and report to the Association at its meeting in Boston, August 3d, 1880. Members of these committees met at Saratoga in August, 1879, and elected Professor J. Taft General Chairman.

The Committees are as follows: of the Southern Dental Association, R. Finley Hunt, Chairman, Washington, D. C.; T. T. Moore, Columbia, S. C.; S. J. Cobb, Nashville, Tenn.; T. S. Waters, Baltimore, Md.; J. R. Walker, New Orleans, La.; F. J. S. Gorgas, Baltimore, Md.

Of the American Dental Convention, R. B. Winder, Ch'n, Baltimore, Md.; J. G. Ambler, New York, N. Y.; F. A. Levy, Orange, N. J.; J. Taft, Cincinnati, O.

Of the American Dental Association, A. W. Harlan, Chairman, Chicago, Ill.; C. N. Pierce, Philadelphia, Pa.; F. H. Rehwinkel, Chillicothe, O.; H. J. McKellops, St. Louis, Mo.

A meeting of chairmen was held in New York, May 11, 1880, at which were present Professor R. B. Winder, Dr. R. Finley Hunt and Professor C. N. Pierce, the latter representing (by proxy) Professor J. Taft, General Chairman. Professor Pierce was also present as a member of the committee of the American Dental Association.

This meeting, in accordance with the powers and duties intrusted to the committees, decided to call a Mass Convention, and fixed the time and place of meeting, Wednesday, August 11, 1880, at 11 o'clock A. M., in the City of New York, for the purpose of organizing a National Dental Association, to be composed of members of every State society in the country.

A Constitution and all necessary regulations will be prepared by a sub-committee, and submitted for revision to a meeting of the full committees, to be held in the same city at 9 o'clock A. M., on Monday, August 9, 1880, at Sturtevant House, so as to be thoroughly prepared for the action of the Mass Convention.

The importance of this measure requires that the whole time of the Convention shall be devoted to the business of organization.

All members of State societies and associations, and those intending to become members, are cordially invited to be present at this Mass Convention and take part in its proceedings.

As this proposed National Association is intended to promote the best interests of the whole Profession of the country, it is important and is especially urged that every State in the Union be represented by as large a number of Dentists as possible.

It is hoped, therefore, that *every* Dentist in the country who can *possibly* go to New York at that time, will be present.

The following Executive Committees have been appointed to act together and make all arrangements for this meeting, such as procuring a hall, hotel accommodations, reduction of railroad fares, etc.

By American Dental Convention, J. G. Ambler, New York, N. Y.; Charles Merritt, New York, N. Y.; H. Townsend, Philadelphia, Pa.; J. H. Smith, New Haven, Conn.; E. D. Fuller, Peekskill, N. Y.; G. A. Mills, Brooklyn, N. Y.

By Southern Dental Association, W. H. Atkinson, New York, N. Y.; S. J. Cobb, Nashville, Tenn.; J. W. Selby, New York, N. Y.

By New York State Dental Society, to co-operate with other committees, O. E. Hill, Brooklyn, N. Y.; L. S. Straw, Newburgh, N. Y.

J. TAFT, General Chairman.
R. FINLEY HUNT, Ch'n Com. So. Dental Ass'n.
R. B. WINDER, Ch'n Com. of A. D. C.

